# Phase II trial of carboplatin and etoposide for patients with recurrent high-grade glioma

**DOI:** 10.1038/sj.bjc.6602105

**Published:** 2004-08-10

**Authors:** E Franceschi, G Cavallo, L Scopece, A Paioli, A Pession, E Magrini, R Conforti, E Palmerini, S Bartolini, S Rimondini, R Degli Esposti, L Crinò

**Affiliations:** 1 Bellaria Hospital, Division of Medical Oncology, Via Altura 3, Bologna 40139, Italy; 2 Department of Human Pathology, Bologna University, Bologna, Italy; 3 Bellaria Hospital, Division of Pathology, Bologna, Italy

**Keywords:** high-grade gliomas, chemotherapy, carboplatin, etoposide, topoisomerase II*α*

## Abstract

We present the results of a phase II trial of carboplatin and etoposide (CE) combination as first-line chemotherapy in patients with recurrent glioblastoma multiforme (GBM) and anaplastic astrocytoma (AA) after surgery and radiotherapy. We assess the activity and the tolerability of this combination. 30 patients with GBM (25) and AA (5) were treated with VP-16 (etoposide) 120 mg m^−2^ and CBCDA (carboplatin) 100 mg m^−2^ for 3 days every 4 weeks. Moreover, we performed a retrospective analysis of topoisomerase II*α* gene status using chromogenic *in situ* hybridisation. The median age was 54 years (21–73 years); Eastern Cooperative Oncology Group performance score was 0-1 in 25 patients and 2 in five patients. All patients had been previously treated with surgical resection (21 radical resections) followed by radiation therapy (40–60 Gy). We observed six (20%) complete responses, three (10%) partial responses and 12 (40%) stable diseases, with a response rate of 30%. The median time to progression was 4 months, while progression-free survival at 6 months was 33.3%. The median survival time was 10 months. Neutropenia occurred in 9 patients: four patients had grade 4, two patients grade 3 and three patients grade 2. In the conclusion of this clinical trial, the CE combination has shown activity in recurrent GBM and AA, with a good toxicity profile. Alterations in the copy number of topoisomerase II*α* gene seem to be a rare event and in our series do not influence response to the CE combination.

Malignant gliomas are a heterogeneous group of brain cancers. For all types, surgery and radiotherapy are considered the standard treatment (Cairncross *et al*, 1994).

Anaplastic astrocytoma (AA) and glioblastoma multiforme (GBM) (World Health Organisation (WHO) grade 3 and 4, respectively) together represent 25–35% of brain tumours and 60% of all gliomas (Russell and Rubinstein, 1989). Despite progress in surgery and adjuvant therapy, patients with high-grade gliomas still have a dismal prognosis (Galanis *et al*, 1998). Repeat surgery may not be feasible because of tumour infiltration into eloquent areas of the brain, and additional irradiation has limited control on further tumour growth and would increase the risk of neurologic toxicity.

Age, histology, extent of surgery and performance status are important prognostic variables for malignant gliomas.

Age significantly affects survival and histology, particularly GBM, which is another important prognostic factor: the median survival from initial diagnosis is 11 months for GBM as compared with 27 months for AA. Furthermore, multiple clinical trials have found performance status, either the Karnofsky performance score or the Eastern Cooperative Oncology Group (ECOG) scale, to be a significant prognostic variable (Wong *et al*, 1999). Recently, the Glioma Outcomes Project data published by Laws *et al* (2003) confirmed that resection (compared with biopsy) is also a strong prognostic factor for survival. Nonetheless, despite the results of the Glioma Outcome Project, it is still not clear if the extent of resection represents an independent prognostic factor or if it is a dependent variable that may reflect resectability rather than resection itself.

Systemic chemotherapy is not considered a standard treatment in high-grade gliomas, but it is usually used in relapsing patients after failure of locoregional therapy.

Carboplatin is an effective alkylating agent in a variety of solid tumours. It has effects with a strong dose–response relationship in malignant glioma cells cultured *in vitro* and there is substantial evidence that carboplatin is one of the most potent cytotoxic agents against human gliomas *in vitro* (Doz *et al*, 1991; Wolff *et al*, 1999). Etoposide is a semisynthetic podophillotoxin that acts against topoisomerase II with demonstrated activity against gliomas and other brain tumours (Fulton *et al*, 1996); furthermore, carboplatin and etoposide cross the blood–brain barrier (Postmus *et al*, 1984; Kiya *et al*, 1992) and have been detected in cerebrospinal fluid.

The topoisomerase II*α* gene is mapped on chromosome 17 and encodes a 170 kDa nuclear enzyme responsible for a cleavage/rejoining reaction of double-strand DNA allowing the separation of intertwined DNA strands. During its catalytic cycle, the enzyme binds covalently to DNA, creating a transient double-strand DNA break. Through this DNA break, the enzyme allows the passage of another DNA double-strand helix. Then, DNA is rejoined and the enzyme dissociates from DNA (Jensen and Sehested, 1997).

Etoposide does not kill cells by blocking the topoisomerase catalytic function, rather it poisons this enzyme by increasing the steady-state concentration of its covalent DNA cleavage complexes: this action converts topoisomerases into physiological toxins that introduce high levels of transient protein-associated breaks in the genome of treated cells (Hande, 1998). There is clear evidence both *in vivo* and *in vitro* of strong synergism between carboplatin and etoposide (Stein *et al*, 1999). A recent study showed the activity of a CBCDA (carboplatin)–VP-16 (etoposide)-based therapy in the treatment of low-grade gliomas in children (Massimino *et al*, 2002). The efficacy of CBCDA or cisplatin (CDDP) in combination with etoposide has been evaluated in various schedules in other trials in high-grade gliomas with various results (Buckner *et al*, 1990; Boiardi *et al*, 1991; Jeremic *et al*, 1992). The role of topoisomerase II*α* in glioma cancer cells is still unclear. Examination of tumour samples has shown that the enzyme is expressed at varying frequency (11–36% of high-grade gliomas, 4% of low-grade gliomas, 7% of medulloblastomas) and in varying amounts (about 2, 4, <20% and 40% in grade 1–4 astrocytomas, respectively). Different groups have tried to correlate the expression of this nuclear protein to anticancer drug response or to prognosis with unclear results: both low and high expression is associated with a favourable prognosis (Dingemans *et al*, 1999; MacGrogan *et al*, 2003). In the past few years, interest in topoisomerase II*α* gene amplification has grown because of correlation with HER2 gene coamplification and with sensitivity to anthracyclines in breast cancer. However, the role of topoisomerase II*α* gene amplification in high-grade gliomas has not, to our knowledge, yet been evaluated. For this reason, we decided to evaluate retrospectively the prognostic and predictive role of topoisomerase II*α* amplification.

The objective of the present study is to assess the activity and the toxicity of the treatment based on the CBCDA and VP-16 regimen in patients with recurrent high-grade glioma previously treated with surgery and radiation therapy. Secondary end points were time to progression (TTP), progression-free survival (PFS)-6, PFS-12 and overall survival (OS).

## PATIENTS AND METHODS

### Patient selection

The eligibility criteria for this study included the following: age ⩾18 years; histological diagnosis of GBM (GBM) or AA; unequivocal recurrent tumour after surgery and radiotherapy or evidence of tumour progression after radiotherapy (a minimal interval of at least 4 weeks from radiotherapy was mandatory); and signed, informed consent.

Other eligibility criteria were ECOG performance status ⩽2, stable corticosteroid dose for at least 2 weeks before study entry, measurable enhancing tumour ⩾1 cm, absolute neutrophil count ⩾1.5 × l0^3^ mm^−3^, platelet count 100 × l0^3^ mm^−3^, serum creatinine level ⩽1.2 mg dl^−1^ and AST level ⩽1.5 times the upper normal limit. Other situations rendering patients ineligible were any chemotherapy for recurrent tumour; a history of malignancy other than *in situ* carcinoma of the cervix or nonmelanoma skin cancer; inability to comply with treatment or follow-up; and pregnancy, lactation or unwillingness to consider effective contraception.

### Treatment plan

For this study, CBCDA plus VP-16 were prescribed as follows: CBCDA (100 mg m^−2^) and VP-16 (120 mg m^−2^) both intravenously each day for 3 days every 4 weeks for a maximum of 12 cycles. Carboplatin was administered by 30-min infusion before etoposide infusion, and then etoposide was sequentially administered by 60-min infusion. Anticonvulsants and antiemetics were prescribed as required. Doses in subsequent cycles were reduced or the interval between cycles lengthened for haematological toxicities. Namely, the doses were reduced by 25% for absolute nadir neutrophil count less than 0.5 × l0^3^ mm^−3^ or platelet count less than 50 × 10^3^ mm^−3^; cycles were delayed until the absolute neutrophil count was at least 1.5 × 10^3^ l^−1^ and the platelet count at least 100 × l0^3^ l^−1^. Treatment was suspended if bone marrow recovery was unsatisfactory after 1 month or in the presence of extrahaematological toxicity grade ⩾3.

### Patient evaluation

All patients have been evaluated for safety and activity according to the intention-to-treat analysis. Treatment was discontinued for progressive disease (PD), in case of unmanageable toxicities, or for any reason at the patient's request.

Pretreatment evaluation included a history and physical examination with documentation of all neurologic symptoms and signs, complete blood cell count, screening biochemistry, chest X-ray and either a CT or MR scan with and without contrast enhancement; all tests were performed within 2 weeks of start of treatment. Eastern Cooperative Oncology Group performance status and corticosteroid dose were recorded at baseline. During treatment, a complete blood cell count was performed weekly. After each cycle of treatment, patients were examined and neurologic symptoms and signs documented, performance status and corticosteroid dose were recorded. Re-evaluation was performed every three cycles with CT or MR scans with the same methodology used at baseline. Disease re-evaluation was anticipated if clinical conditions indicated disease progression.

Response assessment was based on measurable change in tumour size taking into consideration corticosteroid requirements and the neurologic examination. Tumour size was considered the maximum cross-sectional area of the enhancing mass on CT or MR, and calculated by multiplying the largest cross-sectional diameter measured in centimetres by the largest diameter perpendicular to it. Response was defined as follows: complete response (CR), disappearance of all enhancing tumour on a TC or RM scan and neurologically stable or improved, with the patient with steroid off; partial response (PR), ⩾50% decrease in tumour size on a TC or RM scan, corticosteroid dose stable or reduced, neurologically stable or improved; PD, ⩾25% increase in tumour size or any new area of tumour on any follow-up scan, corticosteroid dose stable or increased and neurologically stable or worse; all other situations were considered as SD (Macdonald *et al*, 1990). In assessing response, surgical defects, areas of calcification and nonenhancing abnormalities in these predominantly enhancing tumours were not measured. The response assessment was performed by an independent panel of neuroradiologists.

### Topoisomerase II*α* analysis

The copy number of topoisomerase II*α* was evaluated retrospectively in 27 of 30 tumours by chromogenic *in situ* hybridisation (CISH).

All reagents for the assay were obtained from Zymed Laboratories (South San Francisco, CA, USA) and the manufacturer's suggested method was used. Briefly, 4 *μ*m thick, formalin-fixed, paraffin-embedded sections were baked for 30 min at 60°C and then deparaffinised 10 min in xylene twice and 5 min in ethanol twice. Air-dried tissue sections were placed in a Coplin jar containing Tris-EDTA at 45°C for 20 min, and after a wash in SSC 2 × were pretreated with enzyme (3′ of pepsin 0.4% of HCl 0.1N, heat at 45° for 3′) and after a wash in SSC 2 × were dehydrated.

Denaturation was carried out at 90°C, followed by hybridisation at 37°C for 16 h using a digoxigenin-labelled Topo II*α* probe (Spot-Light™ Topo II*α* probe). After hybridisation, stringency washes and a blocking step were performed. Signals were detected using mouse antidigoxigenin and polymerised horseradish peroxidase-goat anti-mouse followed by development with diaminobenzidine. Slides were counterstained with eosin–haematoxylin 1 : 2 or methyl green 1%. A case of infiltrating ductal carcinoma, with amplified Topo II*α* gene, was used as a positive control and the normal cells with two spots were used as internal positive controls. Topo II*α* oncogene was evaluated by two operators using a Nikon microscope under either a × 20 dry or × 100 oil objective. Amplification was defined as greater than 10 discrete copies per nucleus or as a large gene copy cluster (confluent masses of more than 10 signals) in more than 50% of the nuclei evaluated (Tanner *et al*, 2000): we evaluated at least 400 cells in all cases. Low-level amplification was defined as six to 10 copies per nucleus in more than 50% of cells. Unaltered gene copy was defined as one to five copies per nucleus.

### Statistical methods

A single-stage phase II study was conducted to determine the efficacy of CE combination in the treatment of patients with recurrent high-grade glioma. The targeted accrual goal of 28 patients provided 85% power to differentiate between a response rate (RR) of 10 and 30%. Specifically, the hypothesis that was to be tested was as follows: H_0_: *P*⩽0.1 *vs* H_1_: ⩾0.3, where *P* is the proportion of patients who responded (CR and PR) to treatment.

The study opened in October 1996 and closed in February 2000. For the purposes of statistical analysis and reporting, the following definitions were used: date of diagnosis was the date of the first surgery for a glioblastoma or an AA; date of progression was the date of the first scan documenting ⩾25% increase in tumour size or any new area of tumour or clinical progression (whichever came first); time to initial response was the interval between the inclusion of the patient in the trial and the date of the first scan documenting a ⩾50% decrease in tumour size; time to progression was the interval between the chemotherapy start date and the date of progression. NCI common toxicity criteria were used to score treatment-related side effects. Overall survival was measured from the chemotherapy start date until the date of death or last follow-up examination. Retrospectively, we analysed the amplification of topoisomerase II*α* gene.

The primary end point was the objective RR. Response rates were analysed by Fisher's exact test according to age (<60 or ⩾60 years), resection (gross total resection or partial resection/biopsy), histological type (AA or GBM), ECOG PS (0-1 or 2) and topoisomerase II*α* status (more than two spots per nucleus or diploid). The secondary end points were PFS, OS and the safety and tolerability of treatment. The Kaplan–Meier method was used in the analysis of PFS and of OS. The log-rank test was performed to compare OS and time to progression distributions by age, resection, histology, ECOG PS, response to chemotherapy and topoisomerase II*α* status.

## RESULTS

### Patient characteristics

In all, 30 patients entered the trial; the first began treatment in October 1996 and the last in February 2000. Their pretreatment characteristics, including age, performance status, surgery and dose of radiotherapy are summarised in Table 1

**Table 1:** Pretreatment characteristics of all study patients

**Characteristic**	
*Age (years)*	
Median	60
Range	21–73
	
*Sex*	
Male	18 (60%)
Female	12 (40%)
	
*ECOG PS*	
0	21 (70%)
1	4 (13%)
2	5 (17%)
	
*Surgery*	
Radical	21 (70%)
Partial	9 (30%)
	
*Radiotherapy*	
Yes	30 (100%)
No	0
Range	40–60 Gy
	
*Site*	
Frontal	11 (37%)
Temporal	4 (13%)
Parietal	3 (10%)
Occipital	2 (7%)
Multilobed	10 (33%)
	
*Prior chemotherapy*	
Yes	0
No	30 (100%)

ECOG=Eastern Cooperative Oncology Group.

[Table tbl2]

**Table 2 tbl2:** Topoisomerase II*α* gene status

**Patient number**	**Topoisomerase II*α* gene status**
1	Not done
2	Diploid
3	Diploid
4	Not done
5	More than two spots
6	Diploid
7	Not done
8	Diploid
9	More than two spots
10	Diploid
11	Diploid
12	More than two spots
13	More than two spots
14	Diploid
15	Diploid
16	Diploid
17	Diploid
18	Diploid
19	Diploid
20	Diploid
21	Diploid
22	Diploid
23	Diploid
24	Diploid
25	Diploid
26	Diploid
27	More than two spots
28	Diploid
29	More than two spots
30	Diploid

.

All 30 patients were eligible and evaluable: 25 had glioblastoma and five had AA at the time of surgery. All patients were submitted to surgery followed by radiotherapy. In total, 21 patients (70%) had a macroscopically complete resection and nine patients had partial resection at time of surgery; all patients after surgery were submitted to radiotherapy (range of dose: 40–60 Gy; median: 54 Gy) of the contrast-enhancing lesion (plus a 2-cm safety margin and the area of preoperative edema). The median progression-free interval since completion of radiotherapy (considering both glioblastoma and AA patients) was 4.5 months (range: 2–26 months); this interval was 4 months in glioblastoma patients (range: 2–15) and 7 months in AA patients (range: 3–26). Of note, all but one patients showed relapse after a time ⩾3 months. At the time of recurrence none of the patients had any surgical approach. A total of 25 patients (83.3%, 95% CI 66–93%) had a good ECOG PS score (0-1); and 26 patients (86.7%, 95% 70–95%) were younger than 70 years. The median time between radiotherapy end and chemotherapy was 3 months (range: 40 days–25 months).

### Chemotherapy

After surgery and radiotherapy, all patients with a recurrence or residual disease in progression were treated with a CBCDA (100 mg m^−2^) plus VP-16 (120 mg m^−2^) regimen for 3 days every 4 weeks. All the patients were valuable both for response than toxicity. A total of 115 chemotherapy courses were administered with a median number of three cycles/patient (range: 1–18). We observed 6 (20%, 95% CI 10–37%) CRs, 3 (10%, 95% CI 19–51%) PRs, 12 (40%, 95% CI 25–58%) SD, with a RR (CR+PR) of 30% and a disease control (CR+PR+SD) of 70%. Nine patients (30%, 95% CI 17–48%) had PD.

In the GBM patient group, we observed three (12%, 95% CI 4–30%) CR, three (12%, 95% CI 4–30%) PR and 10 (40%, 95% CI 23–59%) SD, with a RR (CR+PR) of 24% and a disease control rate (CR+PR+SD) of 64%. Nine patients had PD (36%, 95% CI 20–56%).

Univariate analysis showed that RR was significantly correlated with age <60 years old (*P*=0.025) and AA histology (*P*=0.006), while no correlation was found with radical excision, ECOG PS or topoisomerase II*α* status.

### Progression, survival and toxicity

**Figure 1:**
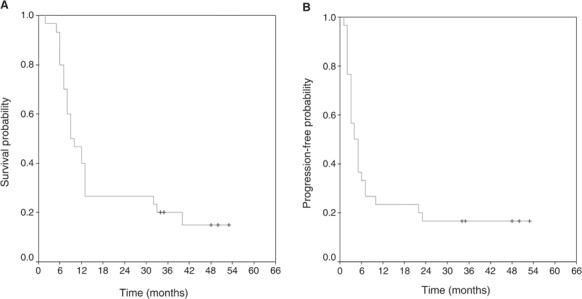
Kaplan–Meier curves for OS (**A**) and TTP (**B**).

At the time of analysis, 26 (86.7%) patients have progressed and 25 (83.3%) patients have died. The median time to progression for all 30 patients was 4 (CI95% 3–5) months, while PFS at 6 months and at 12 months was 33.3 and 26.7%, respectively (Figure 1

). In the GBM subgroup, median time to progression was 3 months, while PFS at 6 months and at 12 months was 20 and 8%, respectively.

The median survival time for the entire group of patients was 10 (95% CI 5–13) months, with 93.3% of patients alive at 6 months, 46.7% at 12 months and 26.7% at 18 months.

The median survival time in the GBM group was 9 months with 92% of patients alive at 6 months, 36% at 12 months and 12% at 18 months.

Survival analysis performed by the Kaplan–Meier method showed that both TTP and OS were significantly correlated with histology (*P*=0.0004 and 0.0007, respectively) and age (*P*=0.014 and 0.0025, respectively), but not with surgical resection grade, ECOG PS and topoisomerase II*α* status.

**Figure 2:**
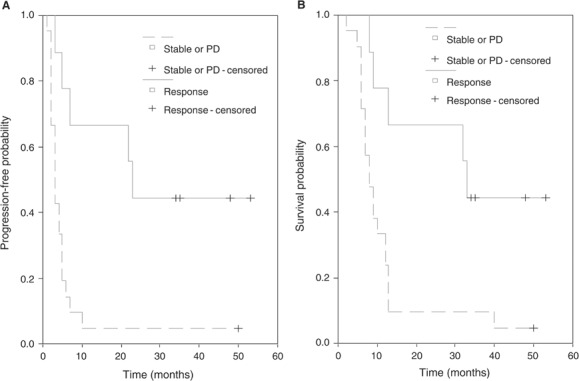
Kaplan–Meier curves for TTP (**A**) and OS (**B**) according to RR (*P*=0.0011 and 0.005, respectively).

Patients who had response to CE chemotherapy showed a significantly longer TTP and survival compared with patients with stable or PD (*P*=0.002 and 0.005, respectively; Figure 2

).

Treatment-related haematological toxicity (according to NCI CTC 2.0) occurred in nine patients with neutropenia: four patients had grade 4, two patients grade 3 and three patients grade 2 (Table 3

**Table 3:** Adverse events reported in patients (*n*=30) during CE (115 cycles)

	**NCI-CTC grade 3 or 4**	
**Adverse event**	**No. of patients**	**%**
Thrombocytopenia	0	0
Neutropenia	6	20
Nausea	0	0
Vomiting	0	0
Constipation	0	0
Ototoxicity	1	3.3

). One patient had severe ototoxicity. No patient had febrile neutropenia or documented infection.

Second-line treatment with temozolomide was given to 11 patients either with disease progression or relapsing after CBCDA-VP16. In this group of patients, no responses were observed and only four patients had SD.

### Laboratory methods

**Figure 3:**
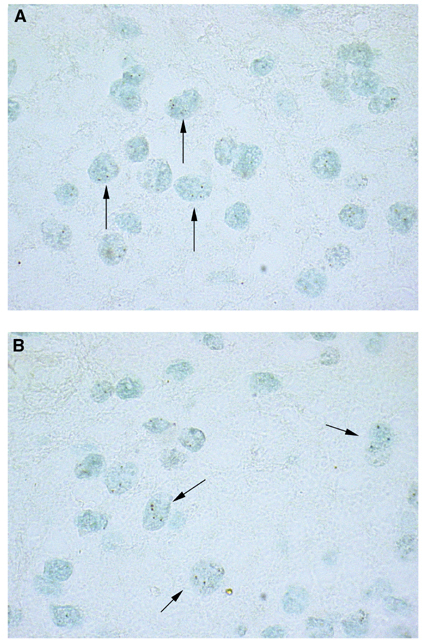
(**A** and **B**) Evidence of more than two dots in the cells as shown by the arrows.

None of the tumours analysed showed amplification of the topoisomerase II*α* gene. As summarized in Table 2, 21 cases showed only two spots per nucleus, while in six tumours the probe revealed more than two spots in only a small percentage of cells (ranging from 5 to 15%), suggesting the presence of clone with polisomy of chromosome 17 (Figure 3A and B

). For statistical analysis, we evaluated if the presence of more than two spots can influence response to CE combination.

Our data indicate that the majority of the cells do not have alterations in the copy number of topoisomerase II*α* gene.

## DISCUSSION

Survival expectations for patients with high-grade gliomas at the time of initial relapse are poor.

Treatment strategies used at the time of tumour recurrence are mainly palliative and include additional surgery, interstitial brachytherapy or radiosurgery and chemotherapy. Unfortunately, many patients are not candidates for additional surgery or radiotherapy. Thus, systemic chemotherapy is often used in these patients as the single modality of salvage therapy. At the time of recurrence different chemotherapy regimens have been used in clinical trials: traditional agents have included nitrosourea drugs (BCNU-CCNU) and procarbazine. More recently temozolomide has been used in clinical studies. Few clinical trials have used carboplatin (CBCDA) and etoposide (VP-16) in the treatment of gliomas (Buckner *et al*, 1990; Jeremic *et al*, 1992; Peterson *et al*, 1996).

Carboplatin is an alkylating agent widely used in the treatment of human tumours, and the synergistic effect when used together with etoposide has been proved either *in vitro* and *in vivo*. Carboplatin has exhibited activity against malignant glioma *in vitro* (Dodion *et al*, 1988), and it has been widely used to treat both paediatric (Gaynon *et al*, 1990; Friedman *et al*, 1992; Gururangan *et al*, 2002) and adult (Poisson *et al*, 1991; Twelves *et al*, 1991; Yung *et al*, 1991; Warnick *et al*, 1994) brain tumour patients.

In the current clinical trial, the CE combination demonstrated activity in patients with recurrent high-grade glioma with an overall RR of 33.3%. The evaluation of activity of this chemotherapy regimen has to be considered in the light of the selection of patients: the majority of patients had a low ECOG PS score and were younger than 70 years, five patients had AA histology (three of these five patients had CR). These findings may explain a favourable response to chemotherapy in our study. A recent study (Braybrooke *et al*, 2003) showed that cisplatin and etoposide phosphate (EP) activity is significantly influenced by topoisomerase II*α* expression levels in patients with advanced breast cancer. In this experience, we evaluated the topoisomerase II*α* gene status without finding any amplification of the topo II*α* gene in our series of tumours. This genomic alteration does not seem to be involved in glioma tumorigenesis. We observed in a few cases (22.2%) an increase in number probably due to polisomy of chromosome 17, but this numeric alteration of topoisomerase II*α* gene copies seems not to influence disease control rate and survival. These data may be due to the low incidence of this alteration found in our series.

In this phase II study, the CE regimen showed a favourable toxicity/activity profile: 10 patients had toxicity and only four of them had grade 4 toxicity with a relevant RR in patients with high-grade gliomas. It is worth noting that we did not use the AUC calculation for the carboplatin individual dosage, and this could be reflected in some underdosages of the drug. However, this regimen should be considered as a good therapeutic option for malignant gliomas and should also be evaluated in more chemosensitive settings such as recurrent oligodendrogliomas.

New approaches to chemotherapy treatment are necessary; biological studies to identify novel therapeutic targets will be useful to find new ways for cancer treatment. The enrolment of patients into rigorous, well-conducted clinical trials, both at tumour diagnosis and after recurrence, will generate new information with regard to investigational therapies, and may offer improved therapies for patients with malignant gliomas.
